# RETRACTED: Kim et al. Hair Cortisol and Fe-BARQ: Evaluating Chronic Stress and Behavior in Cats with Chronic Kidney Disease. *Animals* 2025, *15*, 889

**DOI:** 10.3390/ani16081183

**Published:** 2026-04-13

**Authors:** Ga-Hee Kim, Kyuyoung Lee, Han-Sol Choi, Jin Soo Han, Sun-A Kim

**Affiliations:** 1 Department of Laboratory Animal Medicine, College of Veterinary Medicine, Konkuk University, Seoul 05029, Republic of Korea; rkgml244@konkuk.ac.kr; 2 Department of Microbiology, Institute for Viral Diseases, College of Medicine, Korea University, Seoul 02841, Republic of Korea; wing290@korea.ac.kr; 3 Department of Companion Animal, Shingu College, Seongnam-si 13174, Republic of Korea; hansolc@shingu.ac.kr; 4 Department of Laboratory Animal Medicine, Institute for the 3Rs & Animal Welfare, College of Veterinary Medicine, Konkuk University, Seoul 05029, Republic of Korea; labvet@konkuk.ac.kr; 5 Duffield Institute for Animal Behavior, Department of Clinical Sciences, College of Veterinary Medicine, Cornell University, Ithaca, NY 14853, USA

The journal retracts the article titled “Hair Cortisol and Fe-BARQ: Evaluating Chronic Stress and Behavior in Cats with Chronic Kidney Disease” [1], cited above.

Following publication, concerns were brought to the attention of the Editorial Office regarding the scientific accuracy of the data presented in the published paper [1].

In accordance with the journal’s standard procedures, the Editorial Office and Editorial Board conducted an investigation that confirmed several significant methodological limitations affect the study’s overall validity and reproducibility. The authors cooperated fully with the Editorial Office throughout the investigation and submitted a revised version of the manuscript. However, the Editorial Board determined that the extent of the necessary revisions exceeded what could be addressed through a standard correction. For this reason, Editorial Board have decided to retract the article and invite the authors to submit a revised version, which will be considered as a new submission and subject to the journal’s standard peer-review process.

This retraction was approved by the Editor-in-Chief of *Animals*.

The authors have agreed to this retraction.
